# Estimation of Forces on Actin Filaments in Living Muscle from X-ray Diffraction Patterns and Mechanical Data

**DOI:** 10.3390/ijms20236044

**Published:** 2019-11-30

**Authors:** Srboljub M. Mijailovich, Momcilo Prodanovic, Thomas C. Irving

**Affiliations:** 1 Department of Biological Sciences, Illinois Institute of Technology, Chicago, IL 60616, USA; irving@iit.edu; 2 Department of Chemistry and Chemical Biology, Northeastern University, Boston, MA 02115, USA; momcilo.prodanovic@gmail.com; 3 Bioengineering Research and Development Center (BioIRC), 34000 Kragujevac, Serbia; 4 Faculty of Engineering, University of Kragujevac, 34000 Kragujevac, Serbia

**Keywords:** multiscale model, X-ray fiber diffraction, forces in myofilaments, MUSICO, mechanotransduction

## Abstract

Many biological processes are triggered or driven by mechanical forces in the cytoskeletal network, but these transducing forces have rarely been assessed. Striated muscle, with its well-organized structure provides an opportunity to assess intracellular forces using small-angle X-ray fiber diffraction. We present a new methodology using Monte Carlo simulations of muscle contraction in an explicit 3D sarcomere lattice to predict the fiber deformations and length changes along thin filaments during contraction. Comparison of predicted diffraction patterns to experimental meridional X-ray reflection profiles allows assessment of the stepwise changes in intermonomer spacings and forces in the myofilaments within living muscle cells. These changes along the filament length reflect the effect of forces from randomly attached crossbridges. This approach enables correlation of the molecular events, such as the current number of attached crossbridges and the distributions of crossbridge forces to macroscopic measurements of force and length changes during muscle contraction. In addition, assessments of fluctuations in local forces in the myofilaments may reveal how variations in the filament forces acting on signaling proteins in the sarcomere M-bands and Z-discs modulate gene expression, protein synthesis and degradation, and as well to mechanisms of adaptation of muscle in response to changes in mechanical loading.

## 1. Introduction

Mechanical forces acting in living cells are extremely important in understanding mechanotransduction, cell migration and cell physiological function [1,2,3,4]. Despite their importance, there have been only a few attempts to estimate the magnitude of these forces. For example, Evans et al. measured the magnitude of forces in integrins in adherent cells [5,6,7]. In living cells, the forces in cytoskeletal filaments have been assessed only from observed tension or tractions from mechanical measurements and estimated number of filaments in cross-section [8,9,10,11]. For assessing forces in cell filaments, probably the best test system is striated muscle because it contains well-defined subcellular structure, with established methodologies for measurements of tension and the deformation of actin and myosin filaments by X-ray diffraction. In contracting muscle, X-ray fiber diffraction patterns contain information concerning the position of all molecules within the three-dimensional sarcomere structures as well as the variable local deformation of filamentous structural elements. The simplest method for interpreting the relationship between deformed sarcomere structure, due to developed tension, and meridional reflections in X-ray diffraction patterns, uses the correlation between an average atomic spacing and X-ray patterns along the meridional axis. Using this approach, Huxley et al. (1994) and Wakabayashi et al. (1994) estimated the elastic characteristics of actin and myosin filaments [12,13] by plotting the changes in the axial spacing of the first order actin meridional reflection as a function of developed force. As we show below, however, there is much more information contained in the diffraction patterns if methods can be developed to extract it.

In living cells, fibrous proteins are exposed to mechanical forces interacting with other subcellular structures showing the local strain variations along the fiber length. To account for these variations, a theoretical formalism has been developed for predicting the diffraction patterns from individual helical molecular structures nonuniformly strained along their lengths [14]. Here, we apply this methodology to predict X-ray patterns from contracting muscle with the goal of more precisely interpreting X-ray diffraction patterns from living muscle fibers. We use the Monte Carlo simulation platform, MUSICO [8,15], to generate the geometry of actin filaments deformed by the action of randomly attached myosin heads where, at any instant of time, the distribution of variable strains and intermonomer spacings along myofilaments are available for predicting the X-ray patterns. Adjusting the model parameters in MUSICO to match the X-ray diffraction and mechanical observations of Huxley et al. [12] provides a window to the multiscale relationship between kinetics of individual molecules and the macroscopic mechanical behavior in contracting muscle. In particular, we show that the newly developed MUSICO X-ray diffraction module can predict several orders of actin meridional diffraction reflections where the first order reflection primarily reports the average strains (or monomer spacings) in the filaments, but the higher order meridional reflections also report on the non-uniformity of the spacings [14]. Combining the MUSICO simulations and the observed X-ray diffraction patterns provides, for the first time, the opportunity to assess forces acting in living muscle cells, assuming that the elastic characteristics of actin filaments are known.

## 2. Simulation of X-ray Diffraction Patterns

**Figure 1:**
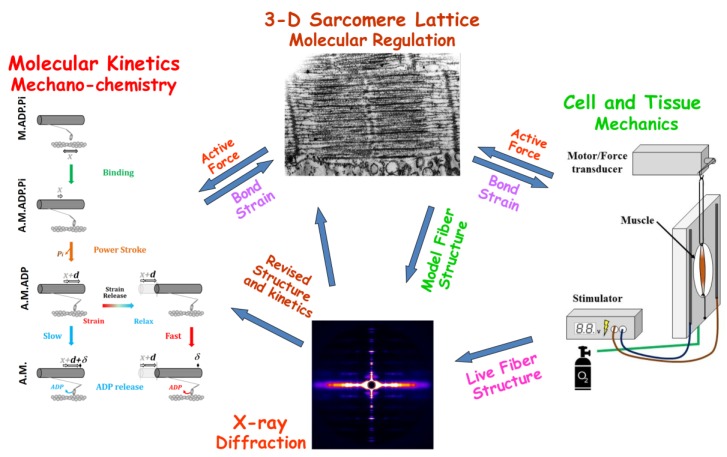
Multiple scale integration of data with the MUSICO computational platform. The model interconnects molecular forces generated by bound crossbridges that, at the level of sarcomere, collectively generate the active force, whereas the equilibrium of external forces and boundary constraints generate the bond strains that modulate crossbridge kinetics [15]. At the level of the sarcomere lattice, regulation by calcium of troponin-tropomyosin interactions with actin modulate crossbridge binding kinetics and force generation, while the bound crossbridges modulate force relaxation when calcium concentration decreases [8]. The MUSICO simulations can predict steady state and transient mechanical responses on external forces and active crossbridge forces. The new module implemented in MUSICO predicts X-ray patterns based on changes in molecular structure in muscle [14]. Comparison of MUSICO predictions of mechanical and structural changes with simultaneous measurements of mechanical responses of muscle fibers, and observations of structural changes at molecular scale by X-ray diffraction in living muscles provides a unique way to test the multiscale models at two scales: molecular and macroscopic.

In order to explain time-resolved X-ray diffraction data, enabled by recent advances in synchrotron small-angle diffraction instruments, a newly developed theoretical approach provides predictions of X-ray diffraction patterns using data from simulations of muscle contraction with 3D spatially explicit models [14]. Specifically, we exploit the computational platform MUSICO, which was originally developed to model muscle mechanics at multiple length scales [8,15], by extending this framework to simulate X-ray diffraction patterns (Figure 1). This combined approach has the power to explain both the structural (X-ray) and the mechanical data simultaneously.

### 2.1. Simulation of Contraction in Muscle Fibers—The MUSICO Platform

The MUSICO platform employs a Monte Carlo algorithm that includes actin-myosin interactions and thin filament regulation in the context of an explicit 3D sarcomere lattice. The simulations can range from a half sarcomere to several sarcomeres in series, in order to predict the observed behavior during force generation, shortening and fast transients [8,15]. The output of the simulation provides a spatially explicit position of each actin, myosin and regulatory protein molecule at any time point following the experimental protocol. By adding the contribution of each molecular interaction individually, including the current state of actin-myosin cycle, weighted by its probability, this ensemble is used to predict the force and length output, that can be matched to observed mechanical data at multiple length scales (Figure 1). The MUSICO simulations predict, for prescribed loading and boundary conditions, crossbridge forces along thick and thin filaments, number and spatial position of bound crossbridges, ATP-ase, Pi and ADP release [8,15,16,17,18,19,20,21]. In addition, the simulations can assess the effect of the compliances of crossbridges, titin, thick and thin filament on molecular interactions and muscle mechanical behavior [8,22].

**Figure 2:**
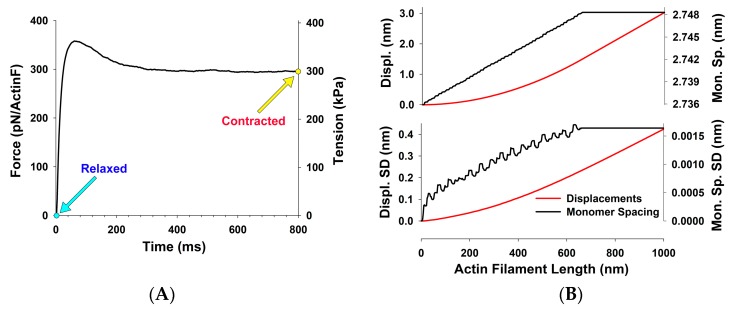
(**A**) The time evolution of force per myosin filament predicted by MUSICO for extensible myosin and actin filaments. The light blue filled circle denotes relaxed muscle where the active force is equal to zero and the yellow filled circle denotes fully developed muscle force at maximal activation. (**B**) At fully developed force, the elongation (displacement) of the actin filament increases approximately quadratically with the length in the overlap region and linearly at non-overlap region (close to the Z-disc). The actin spacing increases approximately linearly following the cumulative increase in force along the overlap region and does not increase in the non-overlap region where force in the actin filament is constant. Due to stochastic binding, there are small step changes in the force and in displacement at the same axial location. The variability of displacement between the actin filaments (i.e., SD) increases along the filaments similarly, but not proportionally, to the mean displacements. The SD of the actin spacing is much more variable and could be up to 12.5% of maximum change of spacings.

By extending MUSICO to generate the meridional X-ray reflections from filamentous actin, using our previously developed theoretical approach (14), where the simulations provide the distribution of forces, strains and the spacings of actin monomers locally along actin filaments, we estimated the mean force on actin filaments in the I band of the sarcomere during isometric force development (Figure 2A). Stochastic spatial 3D binding of myosin to thin filament results in a random stepwise increase, or in rare occasions a decrease, of force along the filaments, thus consequently creating random stepwise changes in the actin subunit spacings. Typical spacing distribution along thin filaments at fully developed isometric tension averaged over 1000 thin filaments, and their standard deviation are shown in Figure 2B. From these distributions, the actin meridional X-ray reflections in contracted muscle can be calculated as shown below.

### 2.2. MUSICO Predictions of X-ray Diffraction Patterns

As described by Prodanovic et al. [14], the actin filament structure is defined, for simplicity, as a discontinuous helix with uniformly or nonuniformly distributed spacings between equivalent subunits along the filaments. The deformed discontinuous helix is represented in a piecewise manner along the filament, where the helix discontinuities are defined at the locations of bound crossbridges in each thin filament. The helical arrangement of subunits in thin filaments also requires that each atom in the subunit follows the same helical path. As the actin monomer contains more than 300 atoms, for simplicity, we used equivalent subunits, representing a group of atoms; in this case, an actin monomer, by the centers of mass and the subunit diameter [14]. The repeating subunits are arranged as a string of points on a continuous helix passing through the subunit’s center of mass forming the equivalent discontinuous helix. Following this approach, we have predicted, using MUSICO simulations, X-ray diffraction patterns from a muscle fiber in the relaxed state, where the actin monomer spacings are uniform, and in the isometrically contracted state where the actin monomer spacings are nonuniform. The resulting diffraction patterns of nonuniformly deformed actin filaments show an increase in the axial widths and reduced intensities of higher order meridional reflections. Thus, the second or higher order actin meridional reflections provide information concerning the degree of spacing nonuniformity along and between actin filaments and, therefore, the degree of the heterogeneity of forces in the thin filaments [14]. The magnitude and distribution of forces in the thin filaments can be achieved by adjusting the input parameters used in the MUSICO simulations, in order to match the observed tension at which the X-ray diffractions are collected.

In this study, we specifically focused on generating X-ray diffraction patterns of nonuniformly deformed thin filaments at fully developed force in isolated living bullfrog sartorius muscle and compared the calculated patterns with experimental X-ray diffraction patterns of Huxley at al. [12]. Here, the effects of nonuniform strains, via nonuniform monomer spacings, and the helix length, corresponding to the length of thin filaments in the sarcomere lattice, are directly reflected in the resulting magnitude and phase for the reflections observed in the diffraction patterns, using a ‘forward problem’ approach. This approach enables correlation of many attributes inherent in MUSICO simulations, for example, the relation of the variability of forces in thin filaments to the observed X-ray reflections. Such analysis is not possible to achieve by direct inverse Fourier transformation of the diffraction data since the X-ray pattern represents the sum of the diffractions from all structures present in the muscle during the X-ray exposure, and the individual contributions cannot be deconvoluted uniquely.

### 2.3. Estimation of Thin Filament Forces in Contracting Muscle

**Figure 3:**
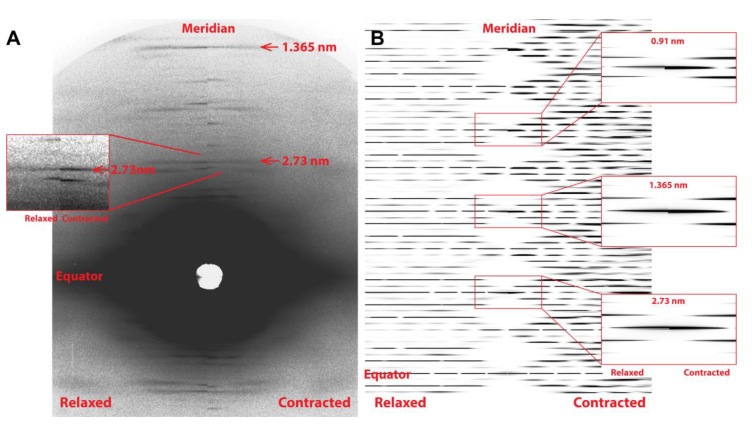
(**A**) 2D X-ray diffraction pattern at rest (Relaxed) and isometrically contracted state (Contracted) from the Experiment No: 2 (2d) [12]. Actin position of first at 2.73 nm and the second at 1.365 nm meridional reflections are marked in by the horizontal red arrows. Enlarged picture of 2.73 nm actin and 2.86 nm myosin meridional reflections shown as an inset to the left. (**B**) MUSICO predicted X-ray diffraction patterns of actin monomer spacing in Relaxed and Contracted muscle. Enlarged 2D patterns of 2.73 nm, 1.365 nm and 0.91 nm actin meridional reflections, at rest and at isometrically contracted state are show as insets to the right.

The actin filament forces are obtained from fitting the intensity distributions for the X-ray actin meridional reflections predicted by MUSICO simulations to the observed X-ray reflections from Huxley et al. [12]. The method includes the following steps: from the observed first and second actin meridional reflections (Figure 3), at approximately 2.73 and 1.365 nm respectively, we (i) estimated the average inter-monomer spacings; (ii) from the average spacing we calculated the average maximum spacing along a representative filament in areas close to Z-disc where the thick-thin filament overlap does not occur (see plateau on right hand side of Figure 2B); (iii) from the maximum monomer spacings, we estimated the average of the maximum forces acting on individual actin filaments. This means actin force is used as guidance for the MUSICO simulations to predict the nonuniform deformation of a large number of actin filaments, calculate the intensity distributions in the actin meridional reflections (first and second order) and compare the calculated reflections to the observations (Figure 3); (iv) by titrating the myosin binding rate to actin, the MUSICO predicted meridional profiles can match actin meridional reflections; and (v) use the MUSICO simulations that matched the observed first and second actin meridional reflection traces to determine the distribution of actin filament forces with the mean values similarly to the estimates determined in steps i–iii.

## 3. Results

The predicted X-ray pattern from a MUSICO simulation, up to the third actin meridional reflection, is shown in Figure 3B which closely resembles the observed pattern shown in Figure 3A. In both panels, the relaxed pattern is on the left and the isometrically contracted pattern is on the right. Contracting muscle has a nonuniform and, on average, longer spacing, which, in the inverse space of the diffraction patterns, is manifested as the meridional reflections move slightly closer toward the equatorial axis. This is clearly seen in the expanded reflection details, on the right of Figure 3B and to the left of the observed reflections in Figure 3A. It is also noticeable that the higher order reflections show larger shifts between the relaxed and contracted patterns, and broader meridional reflection profiles. The shifts are the measure of average change in spacing from relaxed to contracted state, and the widths of the higher order reflections are the measure of the degree of nonuniformity of the actin monomer spacings.

**Figure 4:**
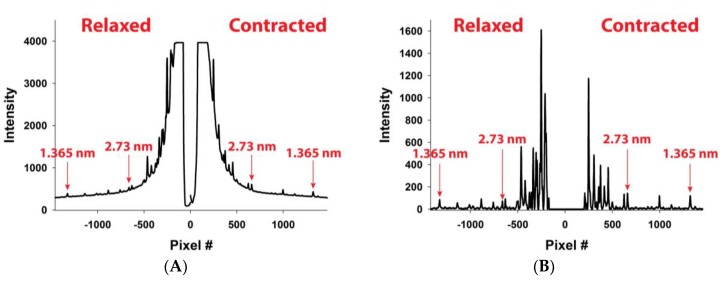
(**A**) Extracted meridional profiles from 2D X-ray patterns of bullfrog sartorius muscle at rest (left side) and at isometrically contracted muscles (right side). The profiles are acquired from the X-ray 2D diffraction images (Experiment No: 2 (2d)) recorded by Huxley et al. [12] and shown in Figure 3A. (**B**) The same patterns after subtracting the background. The arrows point to the 2.73 nm and 1.365 nm first and second order actin meridional reflections respectively. Myosin meridional reflections at 2.86 nm are also visible in the neighborhood of the 2.73 nm reflections.

For assessing forces in the thin filaments, we have chosen five experiments from Huxley et al. (1994) [12], done under isometric conditions where the X-ray patterns (Figure 3A) and meridional actin reflection profiles, up the second order, Figure 4, were available. Each experiment displayed slightly different actin reflection profiles with small but important changes in the mean values of actin monomer spacings, likely caused by the variation in levels of activation between the experiments. For each experiment, we titrated the level of activation via the effective myosin binding rate to actin, $k_{bind}$, in order to match observed first and second actin meridional profiles.

**Figure 5:**
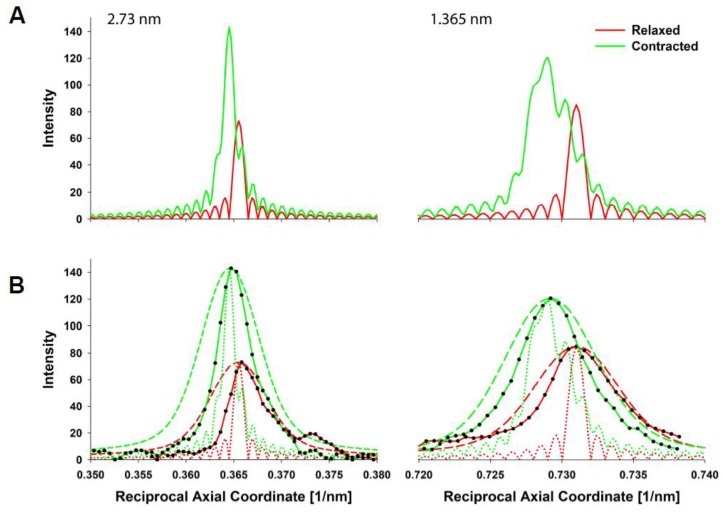
Predicted meridional profiles of muscle in the Relaxed (red line) and Contracted states (sarcomere length: SL = 2.3 μm) (green line). (**A**) MUSICO predicted 1^st^ actin meridional reflections in relaxed and contracted states (left) and predicted 2^nd^ actin meridional reflections (right). Nonuniformity of stretched actin monomers is more easily observed in higher order reflections. (**B**) MUSICO predictions (shown in **A**) corrected for the beam size (dashed lines) are compared to Experiment No: 2 (2d), Huxley et al. [12] data (red and green lines with black circle symbols). Beam dimensions were taken from the experimental setup and convoluted with the MUSICO predicted profiles. The intensity of the predicted profiles is normalized to the observed intensity reported in [12]. The predicted meridional profiles before the correction for the beam size (dotted lines) are shown for comparison.

In Figure 5A, MUSICO predicted profiles for the first and the second reflection are shown, where the solid red lines represent relaxed and the green lines contracted reflection profiles. In order to compare the predicted intensity profiles to the observed, for example, from Experiment No: 2d (Figure 4B), it is necessary to correct for the axial width of the X-ray beam. We, therefore, convoluted the original predicted profiles (solid lines in Figure 5A) with the measured axial size of the X-ray beam to obtain the predicted profiles (dashed lines in Figure 5B) that can be compared to the observed profiles (solid lines with symbols). When we achieved satisfactory fits, the observed X-ray reflections are correlated with MUSICO predicted distribution of spacings, forces in thin filaments and muscle tensions.

**Figure 6:**
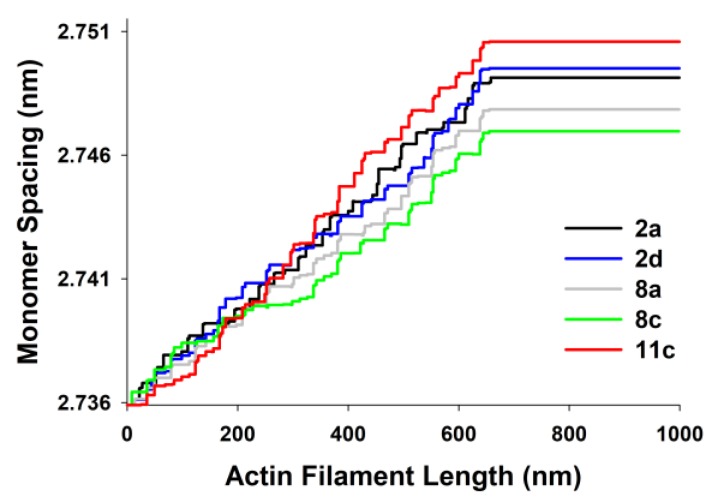
Distribution of subunit spacings along actin filament. Stochastic spatial 3D binding of myosin to actin results in random stepwise increases in force, thus creating random stepwise increases in subunit spacings. The representative monomer spacings are extracted from MUSICO simulations that fitted the profiles of the first and the second actin meridional reflections from the Experiments No: 1–5 (2a, 2d, 8a, 8c and 11c), collected in July 1994 by Huxley et al. [12].

The actin monomer spacings, predicted by MUSICO, as a function of position along the thin filament for the five chosen experiments are shown in Figure 6. The differences are largest in the part of actin filaments in the I band of the sarcomeres (close to Z-discs) and they reflect the changes in observed tensions, on average, between the experiments.

**Figure 7:**
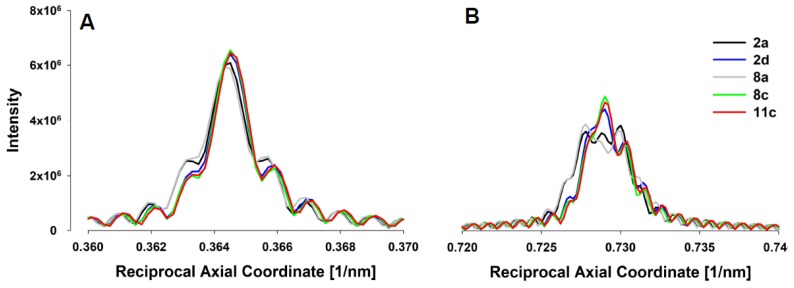
(**A**) 1st actin meridional reflections (~2.73 nm) for 5 different extended actin filaments show subtle differences. (**B**) 2nd actin meridional reflections (~1.365 nm) for 5 different extended actin filaments show notable differences and variations in shape reflecting heterogeneity of spacings along actin filaments and between the actin filaments. The intensity is in arbitrary units coming from the MUSICO simulations and the intensity scale is the same in panels (**A**) and (**B**).

These differences are also present in the predicted integrated intensity profiles of the first and second order actin meridional reflections along the meridian (Figure 7A,B, respectively). The quantitative predictions of the average spacing in relaxed and contracted states, average increase in spacing during the contraction, mean strains, mean actin force and its standard deviation in the I band, and muscle tensions are summarized in Table 1.

**Figure 8:**
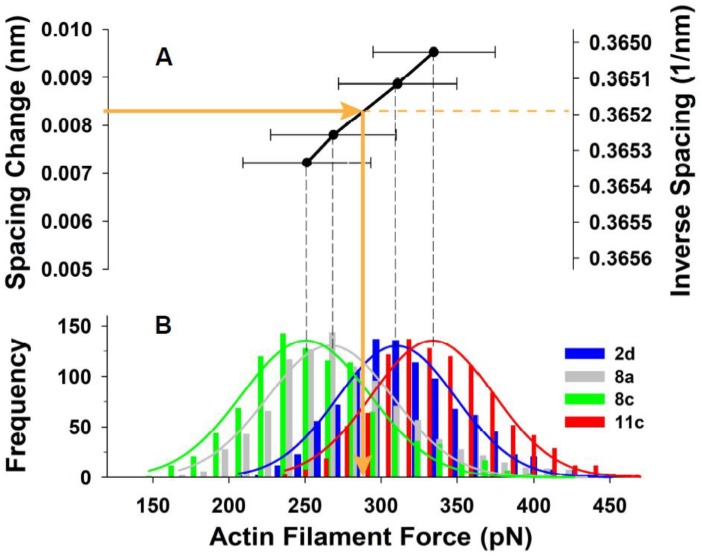
Relationship between force in actin filaments and inverse spacing of a contracted fiber. (**A**) From fitting predicted X-ray meridional diffraction patterns of 2.73 nm and 1.365 nm, we constructed the relationship between the average inverse spacing in contracted state, i.e., spacing change going from the relaxed to the contracted state, and the mean forces in actin filaments. The inverse spacing of the first actin reflection in contracted muscle is shown for reference. (**B**) The histograms of forces in the actin filament (close to the Z-disc) calculated from MUSICO. Here are shown data from four experiments (Experiment No: 2–5 (2d, 8a, 8c, 11c)). Experiment No: 1 (2a) was omitted for clarity because the profile means and distributions were very similar to those observed in Experiment No: 2 (2d). The data, including the estimated mean and variation in actin filament forces, from all five experiments are presented in Table 1.

From the simultaneous fits of mechanical and X-ray data for each experiment we can derive the relationship between the spacing change and mean forces in the actin filaments in the I band, and, importantly, the deviation of the actin forces from the mean (Table 1). The predicted tension shows values in the range from 260 to 340 kPa, that is, not only in the range reported by Huxley et al. (1994) [12], but also in the range between *Rana esculenta* fibers of 228 kPa and in *Rana temporaria* fibers at 4 °C, that is, about ~342 kPa (Linari et al., 1998 [9]). Figure 8A shows the relationship of mean thin filament spacing change (measured from the X-ray patterns) to the mean force (estimated using MUSICO) on the thin filaments for 4 of the 5 individual experiments we analyzed (one experiment was omitted for visual clarity). The horizontal error bars indicate the width of the distributions of forces that gave the mean value in that particular experiment. Figure 8B shows histograms of the calculated force distributions in the individual thin filaments. These force distributions were derived, as described above, using the widths of the second order actin meridional reflections.

## 4. Discussion

The addition of the X-ray diffraction module to the MUSICO platform enables simulations that can predict the forces in actin filaments in living muscles. Moreover, when predictions of these simulations are tightly coupled with spacings and peak shapes of the first and higher order X-ray reflections, considered up to second order in this study, they can provide not only the mean forces on actin filaments as assessed earlier [12,13], but also the variation of the magnitude of these forces, as shown in Figure 8B. The variation of forces in myosin and actin filaments are generated by randomly distributed crossbridges bound to actin, and by using the instantaneous mechanical equilibrium, the MUSICO simulations can provide a view of how these forces vary in time along and between the individual filaments. The magnitude of these variations could be one of the key factors in determining mechanisms of mechano-transduction [23,24,25,26,27]. This information cannot be estimated by any other experimental or analytical technique.

A unique feature of coupling analysis of X-ray diffraction data to MUSICO simulations is that the measurements at the molecular scale, i.e., actin inter-monomer spacings, and macroscopic measurements of tension in live muscle fibers, taken together with multiscale simulations by MUSICO, provide simultaneous model validation at these two length scales using data from the same experiments. Changes in actin monomer spacings, i.e., the observed inverse spacing, and the width of their distributions, as estimated from the width of the second actin reflection (Figure 5B, right panel), are tightly coupled with the mean forces and the variation in the thin filament forces (Figure 8B). The width of the second reflection profile is determined by both the nonuniformity of actin monomer spacings along individual actin filaments and variations between the filaments. The MUSICO simulations can distinguish between these two contributions. These effects could be seen more clearly in higher order actin meridional reflections, as, for example, the third and fourth order reflections observed by Wakabayashi et al. [13]. To obtain data with the higher order actin meridional reflections would require new experiments designed expressly for this purpose.

A key assumption in the estimation of forces in thin filaments is the known elastic characteristics of thin filaments. From mechanical and X-ray diffraction studies, the estimated stiffness of thin filaments in different muscles and preparations, e.g., frog muscles (*Rana esculenta* and *Rana temporaria*), skinned rabbit fibers and in the dogfish muscles, ranges from 67 to about 100 pN/nm per 1 μm long filament [9,28,29,30]. This range of values could be overestimated due to specificities in experimental protocols and complex calculations of the thin filament compliance. The estimated values can depend on species and also differ between fibers from different frog species, for example, *Rana temporaria* vs. *Rana esculenta*. In our MUSICO simulations, we used an actin filament stiffness of ~65 pN/nm per 1 µm F-actin filament from Kojima et al. [31], in preference to other measurements since it was the result of a direct measurement rather than inferred indirectly from mechanical or other data. With Kojima’s stiffness estimate, our model predictions matched well the first and second meridional actin reflection axial spacings in the relaxed and contracting state. In addition, our model predicted the observed tension in Huxley’s experiments. If, on the other hand, we use higher estimates for stiffness (~100 pN/nm) [29,30], the estimated tension from our measurements will be in the range of 450–500 kPa, which are unrealistically high values. The thin filament stiffness from Kojima et al. [31] could still be overestimated because the actin filaments in this study were visualized by phalloidin, and the bound phalloidin could increase the stiffness of the actin filaments [32]. Additionally, recent measurements of Kiss et al. [33] indicated much lower values of thin filament stiffness in mouse soleus muscle and even smaller stiffness in nebulin deficient muscles. A decrease in thin filament stiffness may change the distribution of positions of bound crossbridges along the filaments, the number of bound crossbridges and the average force per filament. The most significant change will be in magnitudes of the mean force per thin and thick filament, where the mean force per thin filament will decrease approximately linearly with the decrease of thin filament stiffness in order to match X-ray observed mean actin monomer spacings. To establish a firm correlation between thin filament spacings and average force per thin filament, it is necessary to measure, beside the X-ray diffraction higher order actin meridional reflections, the muscle tension and the fraction of myofibrils in a given cross-section of the muscle. Kiss et al. observed the first actin meridional X-ray reflection, tension and the occupancy of soleus muscle cross-section [33], but the geometry of the soleus muscle is significantly different from frog sartorius muscle, so their estimates may not be appropriate.

From Huxley’s experimental X-ray patterns used here [12], we estimated a force per actin filament of 297 pN, corresponding to a muscle tension of ~300 kPa. Linearly scaling the estimate for thin filament stiffness from Kiss et al., of 30 pN/nm per 1 μm long thin filament, to Kojima’s stiffness of 65 pN/nm, will bring the estimated tension in Huxley’s experiments on bullfrog sartorius muscle to ~140 kPa. This estimate seems too low, because Huxley et al. reported a range of tensions between 200 and 300 kPa [12]. From similar measurements, Wakabayashi reported muscle tension of ~280 kPa [13]. Assuming that the average tension in Huxley’s experiments is in the range from 250 to 280 kPa, the corrected thin filament stiffness for the stiffening by phalloidin could be in the range of 54 to 60 pN/nm per 1 μm long thin filament, respectively. Taking into account these estimated thin filament stiffnesses, the forces and tensions shown in Figure 8B could be scaled down by 10–15%. If the thin filament stiffness is decreased by 15% (55.25 pN/nm), then the force per actin filament is 251.5 pN and this force can be achieved by reduction in the level of activation, i.e., reduction in apparent $k_{bind}.$

Regardless of the uncertainties discussed above, the mean forces and their variation calculated by simultaneous measurements of X-ray diffraction profiles and muscle tension can be used for the estimation of the number of bound crossbridges, and the mean and variation of the crossbridge forces. This is one of the unresolved fundamental questions in muscle contraction and can be quantitatively assessed by MUSICO simulations employing various crossbridge cycles. For the crossbridge cycle used in MUSICO simulations with parameters appropriate for a muscle tension of 300 kPa, the average number of crossbridges bound is 72.5%, i.e., 108/150; and the number of bound crossbridges may vary between the thick filaments in similar fashion as variation of force shown in Figure 8B. The predicted number of bound crossbridges per myosin filament is similar to the observed 72% of bound crossbridges or 36% of bound heads in frog (*Rana temporaria*) [34]. In the MUSICO simulations, at any moment of time, we have a mixture of weakly and strongly bound crossbridges. Due to the strain dependence, the state transition rates between crossbridge states, each crossbridge experiences different force even in the same state. From this analysis, the estimated average crossbridge force is ~5.5 pN. A decrease in the stiffness of the thin filaments of about 15% requires a decrease in predicted isometric tension also by about 15%, in order to keep average spacing as observed by X-ray diffraction. In the MUSICO simulations, the decrease in muscle force is achieved by reduction in apparent binding rate, $k_{bind}$, i.e., reduced levels of activation; and this decrease is manifested primarily as a decrease in the number of attached crossbridges, to about 61.5% or to 82/150 per half myosin filament with very little change in mean crossbridge force. This estimated crossbridge force is similar to that estimated from simple analysis of mechanical and X-ray observations [9,29,34]. However, crossbridge force estimates from these simple analyses showed a large range of values (from 3.5 to 5.5 pN). This variation in average force could originate in numerous assumptions and simplifications used in their calculation, including rigor vs. fully activated muscle, range of estimates of thick and thin filament stiffnesses, and the range of estimates of crossbridge stiffnesses. Some of thin filament and crossbridge stiffnesses used in previous studies have unreasonable values that can skew the assessed values of the fraction of bound crossbridges and mean crossbridge forces, thus, a comprehensive approach is necessary. The analysis presented here, however, precisely takes into account geometrical factors and requires only a few assumptions regarding the elasticities of the filaments and crossbridges. Importantly, it relates the measured spacings in thin filaments to the forces in actin filaments which vary according to the crossbridge forces. Thus, this approach opens the way toward a more reliable resolution of the enigma of the relative contributions of crossbridge forces and the number of bound crossbridges to the observed tension. The remaining assumptions in the simulations could be removed by constraining MUSICO simulations by multiple experiments on the same muscle type and experimental conditions accompanied with measurements of ATPase and other energetic quantities, along with reliable mechanical measurements of muscle tension accompanying the X-ray diffraction patterns.

The ability to estimate forces in fibrous proteins in living cells could be instrumental in defining the modulation of biochemical processes associated with mechano-transduction. For example, the role of structural proteins in Z-disc and M-band, such as titin, myomesin, obscurin and α-actinin, have been suggested to be involved in active mechano-signaling processes that control gene expression, protein synthesis and protein degradation in the sarcomeres of striated muscle [23,24,25,26,27]. The stochastic nature of myosin binding produces a large variation of the forces exerted by individual myosin filaments on the M-band and actin filaments on the Z-disc. Thus, the signaling functions in muscle mechano-transduction might be related rather to differences in forces between neighboring myosin or actin filaments than to the absolute force acting on the M-band or the Z-disc, respectively. The reason is that M-band and Z-disc structural proteins are deformed by local differences in forces, thus the prospective mechano-transduction process may be associated with fluctuations in forces to which these proteins are exposed. Using X-ray diffraction measurements in concert with MUSICO simulations could be used to define the magnitude of these force fluctuations triggering signaling that modulate gene expression and the adaptation of muscle in response to changes in local mechanical strain.

### Limitations of the Study

Our goal in this paper was to explain, for the first time, how muscle tension and the values of axial actin spacings are related to the heterogeneity of forces in the thin filaments in tetanically contracting muscle. We demonstrated how the magnitude of the heterogeneity of the forces can be estimated using a Monte Carlo approach and the observed higher order meridional reflections. We believe that this goal has been achieved. There are, however, other structural changes in the thin filaments associated with activation that have been observed in muscles stretched beyond filament overlap [13,35], and comparing patterns from resting and actively shortening muscle at velocity close to a maximum [36]. While all studies indicate that at maximum isometric tension, thin filaments are about 0.3% longer than at rest, these latter studies show that when activated without significant binding of force producing heads, thin filaments are about 0.1% shorter and more twisted than at rest. It is likely that consideration of these activation related events could change the results in Figure 8. Our current analysis gives numbers consistent with known forces and filament extensibilities, so these changes are likely to be modest. The mechanisms responsible for these structural changes with activation, however, are not known, so we had no basis on which to attempt to model them using MUSICO. A more precise model of structural changes in the thin filaments in contracting muscle that could address all levels of force and length changes would require new experiments expressly designed for this purpose. Our approach is general enough to address any such new data as they become available.

## 5. Materials and Methods

### 5.1. MUSICO Model Parameters

#### 5.1.1. Sarcomere Geometry and Myofilament Elasticity

The 3D sarcomere lattice in vertebrate striated muscle, is composed of a hexagonal lattice of myosin filaments each surrounded with six actin filaments and each actin filament with three myosin filaments. Each half myosin filament is associated with six titin molecules. The length of the thin (actin) filaments in bullfrog sartorius muscle is ~1 μm, having about 364 monomers in total [37]. The actin filaments have a monomer spacing of ~2.73 nm [12] and the half period of one strand is ~35.5 nm under relaxed conditions [12,13,14]. The length of a myosin filament is ~1.58 μm, having 50 crowns, i.e., 150 myosin molecules per half-thick filament, with a crown spacing of 14.3 nm [38]. This number of crowns and spacing provides maximum overlap with thin filament of ~0.7 μm. The interfilament lattice spacing, $d_{10}$, is 36.5 nm at a sarcomere length of 2.3 μm [39,40]. For simplicity, we limited all stochastic simulations to a half sarcomere with 500 myosin and 1000 actin filaments. This number of filaments is comparable to the number of filaments in a cross-section of a typical myofibril and provides sufficient statistical averaging without running the simulation multiple times.

Actin and myosin filaments are extensible with filament moduli (elastic modulus times cross-section area) derived from X-ray diffraction or direct measurement: for actin, $K_{a}$ = 0.65 × l0^5^ pN; and for myosin $K_{m}$ = 1.32 × 10^5^ pN [12,31].

#### 5.1.2. Three-State Crossbridge Model Parameters

The crossbridge model is defined by a detached state (State 1), a weakly attached or pre-power stroke (State 2) and a post-power stroke state (State 3). Following the approach of Duke [41], the strain dependent state transition rates, as described in Mijailovich et al. [8], are defined by the following parameters: for myosin binding to actin, $\Delta G_{bind}$ = −3 $k_{B}T$ that corresponds to the equilibrium constant is $K_{bind}=k_{bind}/k_{unbind}≅$ 20 and forward rate constant at zero crossbridge strain is $k_{bind}$ = 134 s^−1^ but this average value is adjusted to match anticipated tension in each experiment; for the power stroke, the equilibrium constant is defined by $\Delta G_{stroke}$ = −15 $k_{B}T$, and power stroke d = 10.5 nm; and for ADP release/detachment, $k_{ADP}^{0}$ = 70 s^−1^ associated with the second power stroke δ = 1 nm. The reverse binding rate $k_{13}$ is slow, assumed to be ~0 s^−1^. Because the exponential forms in the expressions for the several state transition rates [8] can become very large and can generate numerical problems—they are capped to $k_{23}^{cap}$ = 1000 s^−1^, $k_{32}^{cap}$ = 100 s^−1^ and $k_{31}^{cap}$ = 10^4^ s^−1^. These values are chosen as optimal values to satisfy Monte Carlo statistics for time steps of the order of 1 μs. When the cap value is reached, the reverse rates are changed to decay exponentially to satisfy the equilibrium constant, $K_{ij}\left(x\right)$ [8,15]. In all simulations, crossbridge stiffness is taken to be κ =1.3 pN/nm (as used by [41]) and the value for $k_{B}T$ = 3.96 pN·nm that corresponds to the temperature of the experiment T = 14 °C = 287.15 °K.

### 5.2. Conversion between Tension and Isometric Force per Actin Filament

The observed actin monomer spacing is directly related to the distribution of forces along the thin filament. Due to the stochastic process of myosin interacting with actin, the forces in the myofilaments fluctuate in time and each filament experiences somewhat different force distributions along the filament. The integrated effect of this heterogeneous distribution of the spacings results in the observed intensity distributions in the actin X-ray reflections. The conversion scale between the average force per filament to tension is related by a factor that takes into account how many actin or myosin filaments there are per unit of the muscle cross-sectional area [9,15]. As the total number of thick filaments in muscle or muscle fiber cross-section does not change with experimental conditions, we used the lattice spacing, $d_{10}$, at slack length, i.e., at the same length where the muscle cross-sectional area has been determined for the estimation of the number of thick filaments per unit of cross-sectional area. This cross-sectional area is used in the tension calculations at any sarcomere length where muscle force is measured. Note that the spacing calculated below is only for the purpose of estimating the number of myosin and actin filaments per unit of cross-sectional area used in calculating observed tension, and this spacing is different than used in MUSICO simulation, where $d_{10}$ corresponds to actual spacing at the sarcomere length of the experiment, i.e., at 2.3 μm. The lattice spacing, in bullfrog sartorius muscle at slack sarcomere length (2.15 μm) is estimated to be $d_{10}$ = 37 nm [39,40]. The number of thick filaments per μm^2^ muscle cross-sectional area, where fraction occupied by myofibrils is taken to be 80% as suggested by Huxley et al. [12], is ~506 half-myosin or ~1012 actin filaments per μm^2^ in a half sarcomere. This scaling factor relates the average thin filament force, over five X-ray diffraction experiments [12], where 297 pN corresponds to a muscle tension of 300 kPa.

### 5.3. X-ray Diffraction Experiments

The experimental data used to test our simulations were extracted from the same diffraction patterns and tension records used for the data published by Huxley and colleagues [12], where complete experimental details are given. The overall experimental arrangement is shown in Figure 1. Briefly, sartorius muscles were dissected from 4–5 inch (10–12.5 cm) bullfrogs and maintained in Ringer’s solution at the desired experimental temperature. X-ray patterns were the sum of 50 ms exposures during the plateau from 20–50 supra-maximally stimulated tetanic contractions (300–600 ms long at 10–14 °C). The same number of 50 ms alternating exposures is taken under resting and during isometric contractions. Patterns were accumulated on the same image plate that was read out at the end of the experiment. Under these conditions, tensions were in the range of 2–3 kg/cm^2^, with the actual values recorded for each experiment. The experiment was stopped when tension dropped to 80–90% of its initial value.

The axial position and the axial width of the first and second order actin meridional reflections were obtained from integrated intensity projections along the meridian. The measured accuracy of the axial reflection profiles was estimated [12] to be to 0.1 pixel out of 600–1300 pixels, so the estimated error would be <0.05%. This is somewhat less than a third of the differences between the inverse spacing changes observed in different muscle samples. This error is in inverse space, thus the error in change of actin monomer spacing is smaller in the higher order meridional reflections. As the peak positions were determined using the centroid of the top half of the meridional peak, the effective error in the peak position is much smaller than 0.1 pixel estimate, i.e., the estimated error would be much smaller than 0.05%, due to smoothing of the shape of the X-ray reflections. Thus, this error is much reduced and has only minor consequence to the calculated estimates of spacings, filament forces and tension.

## 6. Conclusions

The new methodology presented here, combining small angle X-ray diffraction and multi-scale simulation, provides a unique tool for measurement of force acting on a single myosin or actin filament in living muscle fibers. The centroid of the relatively sharp 1st actin meridional peak best describes the average inter-monomer spacing, whereas the much broader width of the 2nd meridional peak reflects both the degree of nonuniformity of spacing along each actin filament and the variation of forces in different actin filaments. MUSICO simulations can separate these two effects on the width and shape of 2nd order actin meridional peak showing that there are large variations in the forces in different actin filaments in a sarcomere during contraction.

The MUSICO simulations generally predicted well the changes in molecular spacings during force development and relaxation in skeletal muscle observed experimentally by H.E. Huxley et al. [12]. The estimated myofilament forces in living muscle fibers provide the possibility for a more precise assessment of crossbridge forces. Moreover, the effect of local differences in myofilament forces acting on signaling molecules in M-band and Z-discs could be important in activating protein synthesis and degradation, and triggering muscle adaptation in response to changes in mechanical strain.

This approach can be extended to any muscle system, and it could ultimately provide an interpretive framework for studying mechanisms of inherited or acquired muscle diseases in transgenic animal models and, prospectively, humans. By extracting the maximum information from the X-ray patterns, in combination with the physiological data, this approach provides a template to test hypotheses concerning crossbridge and regulatory protein action in working muscle.

## Figures and Tables

**Table 1:** Estimated mean forces on actin filaments close to the Z-disc and SD from 5 experimental diffraction patterns. From inverse spacing peaks at ~2.73 nm and ~1.365 nm, we calculated the change in the spacing between the contracted and relaxed states (Del H). Calculated patterns using MUSICO are compared to the experimental peaks at ~2.73 nm and ~1.365 nm to match the position of the peaks (in inverse space) and their widths. Good matches of the calculated to the experimental X-ray peak widths allow estimation of the mean forces and their SD in the actin filaments close to Z-disc. The estimated mean tension is calculated from the mean actin force per myosin filament assuming $d_{10}$ = 37 nm and 80% of cross-section occupied by myofilaments at slack sarcomere length of 2.15 μm. The individual experiments are labeled as numbers (1–5), and the original identifiers are put in parentheses in order to allow comparison to the original paper by Huxley and colleagues (12).

Exp. No:	Relax	Isometric	Del H	Strain	Mean Force	SD	Tension
	nm	nm	nm	%	pN/ActinF	pN/ActinF	kPa
1 (2a)	2.73015	2.73893	0.00878	0.322	309.7	41.1	313.4
2 (2d)	2.73037	2.73924	0.00887	0.325	312.8	38.9	316.6
3 (8a)	2.73234	2.74014	0.00780	0.285	274.9	41.3	278.2
4 (8c)	2.72991	2.73713	0.00722	0.264	254.7	42.0	257.8
5 (11c)	2.73171	2.74124	0.00953	0.349	335.9	39.9	340.0

## Abbreviations

3D	Three dimensional
2D	Two dimensional
MUSICO	Muscle Simulation Code
ATP	Adenosine tri-phosphate
ADP	Adenosine di-phosphate
Pi	Inorganic phosphate
ATP-ase	Rate of ATP consumption
ActinF	Actin filament
SD	Standard deviation
